# Perceptions and use of electronic cigarettes among young adults in China

**DOI:** 10.18332/tid/102788

**Published:** 2019-03-04

**Authors:** Xinsong Wang, Xiulan Zhang, Xiaoxin Xu, Ying Gao

**Affiliations:** 1 School of Social Development and Public Policy, Beijing Normal University, Beijing, China

**Keywords:** smoking cessation, China, young adults, perception, electronic cigarette

## Abstract

**INTRODUCTION:**

Little is known about the perception and use of e-cigarettes by the Chinese, particularly the young people. This study reveals the awareness, attitudes, and use of e-cigarettes among young adults in China, examines the relationship between smoking behavior and e-cigarette perception and use, and demonstrates the phenomenon of e-cigarette gifting.

**METHODS:**

We used results from a mobile app-based survey conducted in November 2015 that included 10477 young Chinese adults aged between 19 and 29 years. Bivariate tests were conducted to analyze perception and use of e-cigarettes by respondents of different smoking status. Multivariate logistic regressions were applied to examine the correlates of e-cigarette use and perception and e-cigarette gifting behavior, particularly the factors of tobacco smoking status and quitting behavior.

**RESULTS:**

Among the surveyed young adults, 88.40% were aware of e-cigarettes, and nearly a quarter of all respondents had used e-cigarettes by the time of our survey. Multivariate regression results demonstrated that current smokers with quitting experience were more likely to be aware of and to use e-cigarettes than current smokers with no quitting experience. Smokers with quitting experience also were more inclined to promote e-cigarettes to others by either recommending them or giving them as gifts.

**CONCLUSIONS:**

E-cigarettes have gained popularity among young adults in China and smokers, especially those who had tried quitting, were more likely to have known and used e-cigarettes. More empirical research on the relationship between e-cigarette use and smoking cessation is warranted to better inform a potential regulatory framework in China.

## ABBREVIATIONS

E-cigarettes: electronic cigarettes
CATS: China Adult Tobacco Survey
US: United States of America
UK: United Kingdom of Great Britain and Northern Ireland
ITC: International Tobacco Control

## INTRODUCTION

Electronic cigarettes (e-cigarettes) are electronic devices that heat a liquid solution that typically contains glycerol and propylene glycol, with or without nicotine and flavours, and emit aerosols inhaled by users^1^. E-cigarettes have become increasingly popular worldwide in recent years, and the entry of the tobacco industry in the e-cigarette market has caused more concern within the public health community^2^.

China produces more than 80% of the world’s e-cigarettes^3^. About 500 physical stores and nearly 7000 online stores on Taobao, one of the largest e-commerce sites in China, sell e-cigarettes in the country^4^. Research on e-cigarette-related attitudes and behavior among Chinese is burgeoning but still limited, especially for young adults. One study using data from the 2015 Global Youth Tobacco Survey showed that 45.0% of middle school students had heard of e-cigarettes, but only 1.2% reported using e-cigarettes in the last 30 days^5^. A 2015 online survey in China showed that 89.52% of adolescents aged 12– 18 years were aware of e-cigarettes while the ever use rate was 26.44%^6^. According to the 2015 China Adult Tobacco Survey (CATS), 40.5% of adults aged 15 years and above had heard of e-cigarettes and 3.1% had ever tried them^7^.

Certain characteristics of young adults make the age group particularly interesting to study regarding their perceptions and use of e-cigarettes. Generally speaking, young adults may be more likely to try new things, and they are more able than adolescents to afford e-cigarettes, which are more expensive than cigarettes on average. According to a study that reviewed 48 earlier studies that reported a correlation between age and e-cigarette awareness and use, older adolescents and young adults demonstrated ‘driving levels of awareness and use of e-cigarettes’^8^. Young adults in the 2015 CATS and the 2013–2014 China City Adult Tobacco Surveys had a higher percentage of e-cigarette awareness and use^7,9^. Surveys in the US in 2012–2015 showed that young adults (18–24 years old) were also less likely than other groups to perceive e-cigarettes as equally or more harmful than conventional cigarettes^9^.

E-cigarette awareness and use are found to be correlated with smoking behavior. Surveys of US households showed that current and former smokers were significantly more likely than non-smokers to be aware of e-cigarettes^8^. Smoking status and frequency of cigarette consumption among US adolescents were correlated with frequency of e-cigarette use^10^. A study of European Union adults showed that being current and former smokers were the strongest correlates of using e-cigarettes, and current e-cigarette users reported high rates of smoking cessation and reduction^11^. The 2014 National Health Interview Survey in the US showed that daily e-cigarette use was most common among adults who recently quit smoking cigarettes^12^.

Existing research findings on sociodemographic correlates of e-cigarette use by all age groups are mixed. For example, one study using data from Wave 8 of the International Tobacco Control (ITC) Four-Country Survey conducted July 2010 to June 2011 in the US, Canada, Australia and the UK found that younger, better educated, and higher-income respondents were more likely to be aware of e-cigarettes, while younger, female respondents were more likely to have ever tried e-cigarettes^13^. But a study based on the 2009–2010 data in the US showed that, although less educated respondents were more likely to have heard of e-cigarettes, they also were more likely to have tried e-cigarettes than better educated groups^14^. Still, data derived from the ITC Netherlands Survey in 2014 demonstrated that income and education level did not matter in ever trial of e-cigarettes^15^. As for China, a previous study showed that among urban male smokers, the likelihood of being current e-cigarette users was significantly higher among smokers aged 15–29 or 30–49 years than those aged over 50 years, and among those with higher education than those with below high school education^9^.

In terms of motivations for using e-cigarettes, some studies have shown that users considered e-cigarettes helpful in reducing or quitting smoking, a safer and healthier alternative to conventional cigarettes, to have better and more flavors, fashionable, and convenient in places where smoking bans applied^13,15-17^. Other studies have found that alcohol use^18,19^ and peer influence^20-22^ were associated with higher rate of e-cigarette use. Still, some research has found that people use e-cigarettes simply out of curiosity or ‘just because’^17,23^.

In the Chinese context, e-cigarette use also may be related to the social norm of gifting-based relationships. Cigarette gifting and sharing among friends and colleagues has long been common practice for Chinese smokers and seen as an important way of maintaining social networking and relationships^24^. Existing studies have shown that the culture of cigarette gifting has promoted smoking behavior among Chinese youth^25^ and prevented successful smoking cessation^26,27^. As e-cigarettes become more prevalent, the culture of e-cigarette gifting (giving e-cigarettes to friends, family members and others) may be an important characteristic, particularly among young people.

This study is one of the first to map the landscape of e-cigarette use among young adults in China. Using an online survey covering most of the provinces, we collected previously unknown information about e-cigarette use in a country that produces most of the world’s e-cigarettes and has the largest smoking population in the world. By focusing on the young adults aged 19–29 years, we provide a picture of how widely e-cigarettes are known and used, who are more likely to have used e-cigarettes, their motivations, and the phenomenon of e-cigarette gifting.

## METHODS

### Participants and procedure

We conducted a mobile app-based survey between 16 and 18 November 2015. Our questionnaires were pushed to the mobile devices of 152622 registered users of Survey Baby (*wenjuan bao*), a mobile app developed by the Chinese public opinion research company, Kdnet Cloud Intelligence System.

A time limit of 48 hours was set for responses, and 22081 people under the age of 40 years answered the questionnaires within the time frame. The app system was able to detect the locations of respondents through their internet protocol addresses and global position system of their devices so only users in China were included. All submitted questionnaires went through a systemic procedure that checked on their completeness, sociodemographic consistency with the registered information, and whether time spent on each question was reasonable (the minimum time for each question was expected to be 3 seconds). A second round of logic checks was performed to focus on smoking history, and perception and use of e-cigarettes.

In total, 17663 or 80% of the respondents had complete and valid answers. Each was compensated $0.80 to their account. Of the valid respondents, 2042 or 11.56% were between the ages of 12 and 18 years, 10477 or 59.32% were aged between 19 and 29 years, and 5144 or 29.12% were aged between 30 and 39 years. This study analyzes the use of e-cigarettes and its correlates among the 10477 young adults (age group 19–29 years).

### Measures

The respondents were divided by their tobacco smoking status into current smokers, former smokers (those who had quit), and never smokers. The current smokers were asked whether they ever tried to quit smoking (yes vs no).

We first measured respondents’ awareness of e-cigarettes with the question: ‘Have you heard of e-cigarettes?’ (yes vs no). Those who answered ‘yes’ were further asked: ‘Where have you heard of e-cigarettes?’. All respondents were asked about their perceptions of e-cigarettes. The choices were: ‘Harmful to users’, ‘Harmful to others’, ‘Addictive’, and ‘Safe replacement for conventional cigarettes’.

We measured the use of e-cigarettes with the question: ‘Do you use e-cigarettes now?’ and the choices were ‘Often (≥20 times)’, ‘Occasionally (<20 times)’, ‘Ever used (1–2 times)’, and ‘Never used’. We further asked those who had used e-cigarettes how they obtained the products, their reasons for using them, and the factors considered when choosing e-cigarettes. The options on how they obtained e-cigarettes included the internet, physical stores, TV shopping, received as gifts, and other. The options on the reasons of using e-cigarettes included avoiding the harm of cigarettes, avoiding secondhand smoke for others, helping to quit smoking, e-cigarettes being cheaper than cigarettes, avoiding smoking bans, and being fashionable. The options for factors considered in selecting e-cigarettes included brand, price, taste, vapor level, battery power, nicotine level, and product design.

Finally, we asked the respondents whether any of their family members or friends used e-cigarettes, whether they used other substances such as alcohol, and whether they ever recommended e-cigarettes to others or sent e-cigarettes as gifts to others. The questionnaire also included a number of sociodemographic questions about gender, education, economic status, employment status, and type and location of household registration (*hukou*).

### Analysis

SAS 9.3 statistical software was used for the analysis. We first generated descriptive statistics on the variables of e-cigarette perception, use, and promotion, and then conducted bivariate tests to analyze e-cigarette perception and use by respondents of different smoking status. Finally, multivariate logistic regressions were applied to examine the correlates of e-cigarette awareness and use, and the correlates of e-cigarette gifting.

We applied four logistic regressions to estimate the correlates of both awareness (Models 1 and 2) and use of e-cigarettes (Models 3 and 4). For Model 1, a major correlate variable was cigarette smoking status (current, former, versus never smoker). Model 2 also compared current smokers who had tried to quit and those who had not tried to quit with never smokers. Also in the model were some sociodemographic variables including gender (male vs female), economic status (very wealthy, comfortable, fair, difficult, versus very difficult), education level (junior college, college, postgraduate, versus high school or lower), marital status (married, never married, versus other), employment status (full-time, part-time, versus unemployed), and type and location of birth registration (*hukou*) (urban versus rural, and local versus migrant).

In Models 3 and 4, we generated a dichotomous variable for e-cigarette use by coding those respondents who never used e-cigarettes as 0, while all others were coded as 1, no matter how often they used them. A dichotomous variable would help us to more straightforwardly examine the correlation of e-cigarette use with sociodemographic, perception and smoking status variables. The major correlate variables were smoking status (current, former, versus never smoker), perception of e-cigarettes (not harmful versus harmful, no secondhand smoke harm versus secondhand smoke harm, not addictive versus addictive, and safe replacement for cigarettes versus not), alcohol use (often, occasional, seldom drink, versus never drink), use of e-cigarettes by family members (yes vs no), as well as the sociodemographic characteristics. Model 4 also compared current smokers who had tried to quit and those who had not tried to quit with never smokers.

We also conducted four logistic tests to examine the correlation between e-cigarette promotion and cigarette smoking status, e-cigarette perception, alcohol use, use of e-cigarettes by family members, and sociodemographic variables. Models 1 and 2 tested the correlates of sending e-cigarettes as gifts, while Models 3 and 4 tested the correlates of recommending e-cigarettes to others.

## RESULTS

### Descriptive statistics

Among the 10477 young adults in our sample aged 19–29 years, 3327 or 31.76% were current smokers, 1124 or 10.73% were former smokers, and 6026 or 57.52% had never smoked. The current smoking rate reported for males was lower than that reported by a recent study while that for females was much higher. Our study shows that 44.96% of males and 11.33% of females were current smokers, compared to 52.1% and 2.7% (aged 15 years and above), respectively, reported in the Chinese Center for Disease Control and Prevention study^28^. Table 1 presents the sociodemographic characteristics of the respondents by their smoking status.

**Table 1:** Sociodemographic characteristics by tobacco smoking status (N=10477)

*Characteristics*	*Total sample (N=10477) % (n)*	*(Urrenl smokers (N=3327) % (n)*	*Former smokers (N=1124) % (n)*	*Never smokers (N=6026) % (n)*
**Gender**				
Male	60.74 (6364)	85.99 (2861)	74.64 (839)	44.21 (2664)
Female	39.26 (4113)	14.01 (466)	25.36 (285)	55.79 (3362)
**Economic status**				
Very wealthy	2.67 (280)	4.45 (148)	4.00 (45)	1.44 (87)
Comfortable	35.25 (3693)	38.65 (1286)	38.70 (435)	32.72 (1972)
Fair	52.39 (5489)	47.94 (1595)	48.84 (549)	55.51 (3345)
Difficult	6.72 (704)	6.43 (214)	6.05 (68)	7.00 (422)
Very difficult	2.97 (311)	2.52 (84)	2.40 (27)	3.32 (200)
**Education level**				
High school or lower	18.22 (1909)	21.52 (716)	16.19 (182)	16.78 (1011)
Junior college	38.66 (4050)	40.58 (1350)	39.77 (447)	37.39 (2253)
College	38.80 (4065)	34.17 (1137)	37.81 (425)	41.54 (2503)
Post-graduate	4.32 (453)	3.73 (124)	6.23 (70)	4.30 (259)
Employment status				
Full-time employment	46.64 (4886)	58.04 (1931)	46.89 (527)	40.29 (2428)
Unemployed	30.70 (3216)	20.77 (691)	26.87 (302)	36.89 (2223)
Part-time employment	22.67 (2375)	21.19 (705)	26.25 (295)	22.82 (1375)
**Marital status**				
Married	27.21 (2851)	28.04 (933)	24.73 (278)	27.22 (1640)
Divorced/separated	3.20 (335)	4.06 (135)	7.03 (79)	2.01 (121)
Never married	63.80 (6684)	62.88 (2092)	60.94 (685)	64.84 (3907)
Other	5.79 (607)	5.02 (167)	7.30 (82)	5.94 (358)
**Location and type of hukou (household registration)**				
Urban/local	29.82 (3124)	32.52 (1082)	29.72 (334)	28.34 (1708)
Urban/non-local	12.27 (1286)	13.23 (440)	16.81 (189)	10.90 (657)
Rural/local	40.42 (4235)	38.32 (1275)	37.10 (417)	42.20 (2543)
Rural/non-local	17.49 (1832)	15.93 (530)	16.37 (184)	18.55 (1118)

Table 2 shows the awareness, perception and use of e-cigarettes by the respondents. In general, Chinese young adults were highly aware of e-cigarettes (88.40%), especially among current and former smokers (95.34% and 91.81%, respectively). Even 83.94% of never smokers had heard of e-cigarettes. The internet was the top source of learning about e-cigarettes in China (45.11%) and more so for smokers than for never smokers, followed by TV (28.78%), and family members or friends (16.51%).

**Table 2:** Awareness, perception and use of e-cigarettes by tobacco smoking status (N=10477)

*Characlerislics*	*Tolal sample (N=10477) % (n)*	*Currenl smokers (N=3327) % (n)*	*Tinner smokers (N=1124) % (n)*	*Never smokers (N=6026) % (n)*	*X^2^*	*p*
**Aware of e-cigarettes**	88.40 (9262)	95.34 (3172)	91.81 (1032)	83.94 (5058)	286.3	0.0001
**Source of awareness**						
Internet	45.11 (4178)	48.90 (1551)	47.97 (495)	42.15 (2132)		
TV	28.78 (2666)	26.70 (847)	27.33 (282)	30.39 (1537)		
Newspaper	2.83 (262)	3.91 (124)	4.75 (49)	1.76 (89)	121.8	0.0001
Outdoor advertisement	3.56 (330)	3.25 (103)	3.78 (39)	3.72 (188)		
Family or friends	16.51 (1529)	13.62 (432)	14.05 (145)	18.82 (952)		
Others	3.21 (297)	3.63 (115)	2.13 (22)	3.16 (160)		
**E-cigarettes harmful to users**						
Yes	27.77 (2909)	26.57 (884)	26.78 (301)	28.61 (1724)		
No	27.93 (2926)	33.48 (1114)	35.59 (400)	23.43 (1412)	151.4	0.0001
Don't know	44.31 (4642)	39.95 (1329)	37.63 (423)	47.96 (2890)		
**E-cigarettes harmful to others**						
Yes	22.67 (2375)	20.53 (683)	22.15 (249)	23.95 (1443)		
No	36.36 (3809)	41.84 (1392)	44.75 (503)	31.76 (1914)	137.8	0.0001
Don't know	40.98 (4293)	37.63 (1252)	33.10 (372)	44.29 (2669)		
**E-cigarettes addictive**						
Yes	25.16 (2636)	19.48 (648)	21.44 (241)	28.99 (1747)		
No	34.23 (3586)	42.23 (1405)	47.15 (530)	27.40 (1651)	324.5	0.0001
Don't know	40.61 (4255)	38.29 (1274)	31.41 (353)	43.61 (2628)		
**E-cigarettes a safe replacement for cigarettes**						
Yes	40.02 (4193)	44.18 (1470)	42.26 (475)	37.31 (2248)		
No	29.85 (3127)	29.13 (969)	34.70 (390)	29.34 (1768)	89.5	0.0001
Don't know	30.13 (3157)	26.69 (888)	23.04 (259)	33.36 (2010)		
**Use of e-cigarettes**						
Often (> 20 times)	2.34 (245)	5.08 (169)	4.54 (51)	0.41 (25)		
Occasionally (<20 times)	9.28 (972)	18.64 (620)	21.89 (246)	1.76 (106)	2954.4	0.0001
1-2 times	12.83 (1344)	28.13 (936)	21.26 (239)	2.80 (169)		
Never used	75.56 (7916)	48.15 (1602)	52.31 (588)	95.02 (5726)		
**Sources of e-cigarettes**						
Internet vendors	43.19 (1106)	47.01 (811)	35.26 (189)	35.33 (106)		
Licensed cigarette stores	33.85 (867)	32.06 (553)	40.67 (218)	32.00 (96)		
TV shopping channel	10.74 (275)	9.22 (159)	13.99 (75)	13.67 (41)	53.4	0.0001
Received as gifts	6.64 (170)	6.14 (106)	6.16 (33)	10.33 (31)		
Other sources	5.58 (143)	5.57 (96)	3.92 (21)	8.67 (26)		
**Reasons for using e-cigarettes**						
Avoid the harm of cigarettes	39.24 (1005)	41.97 (724)	36.57 (196)	28.33 (85)	22.0	0.0001
Avoid the secondhand smoke harm	43.46 (1113)	42.61 (735)	47.39 (254)	41.33 (124)	4.43	0.1093
Help to quit smoking	41.16 (1054)	48.29 (833)	41.23 (221)	0.00 (0)	246.1	0.0001
Cheaper than cigarettes	13.67 (350)	13.80 (238)	12.50 (67)	15.00 (45)	1.1	0.5783
Avoid smoking bans	13.63 (349)	15.01 (259)	12.13 (65)	8.33 (25)	11.0	0.0041
Fashionable	11.01 (282)	11.59 (200)	6.72 (36)	15.33 (46)	16.4	0.0003
Out of curiosity	20.07 (514)	19.83 (342)	15.30 (82)	30.00 (90)	26.1	0.0001
**Factors in selecting e-cigarettes**						
Brand	33.70 (863)	33.16 (572)	35.82 (192)	33.00 (99)	1.4	0.5040
Price	38.46 (985)	40.29 (695)	37.13 (199)	30.33 (91)	11.2	0.0037
Taste	47.36 (1213)	51.13 (882)	43.84 (235)	32.00 (96)	40.9	0.0001
Vapor level	33.11 (848)	34.55 (596)	31.72 (170)	27.33 (82)	6.6	0.0368
Battery power	15.78 (404)	16.00 (276)	16.23 (87)	13.67 (41)	1.2	0.5617
Nicotine level	24.76 (634)	25.22 (435)	23.32 (125)	24.67 (74)	0.8	0.6733
Product design	12.22 (313)	13.22 (228)	9.51 (51)	11.33 (34)	5.5	0.0647
Family/friends using e-cigarettes	36.36 (3809)	46.89 (1560)	44.93 (505)	28.94 (1744)	338.4	0.0001

Compared with never smokers, both current and former smokers were more likely to perceive e-cigarettes as being not harmful to users (33.48% and 35.59, respectively) and to others (41.84% and 44.75%, respectively). In contrast, 23.43% and 31.76% of never smokers perceived e-cigarettes as being not harmful to users or others, respectively. Also, current and former smokers were much more inclined to perceive that e-cigarettes were not addictive (42.23% and 47.15%, respectively) than never smokers (27.40%), and they were more likely to believe that e-cigarettes were a safe replacement for conventional cigarettes (44.18% and 42.26%, respectively). But surprisingly, for all the questions regarding the harm of e-cigarettes, more than one-third of respondents across the board answered ‘Don’t know’, suggesting an overall lack of knowledge on e-cigarette products.

The usage rate of e-cigarettes among Chinese young adults was 24.45%, although only 2.34% of them were frequent users (≥20 times). More than half of current smokers and nearly half of former smokers had tried e-cigarettes, but only 5% of never smokers had done so. The internet, physical stores and TV shopping channels were the main sources for young adults to obtain e-cigarettes, and 10.33% of the never smokers obtained e-cigarettes as gifts, compared with about 6% for smokers.

The top reasons for using e-cigarettes were to avoid health harms both to themselves (39.24%) and to others (43.46%) and to help quit smoking (41.16%). Interestingly, 30% of never smokers picked up e-cigarettes out of curiosity while fewer current or former smokers did it for the same reason. When selecting e-cigarettes, the respondents paid more attention to brand (33.70%), price (38.46%), taste (47.36%), vapor level (33.11%), and nicotine level (24.76%). It seems that smokers were more sensitive to the price and taste of e-cigarettes than never smokers.

Finally, current and former smokers also were more likely (46.89% and 44.93%, respectively) than never smokers (28.94%) to have family members or friends who had used e-cigarettes. As seen in Table 3, current and former smokers also were twice as likely as never smokers to have bought e-cigarettes as gifts, and more likely to have recommended e-cigarettes to others.

**Table 3:** E-cigarette promotion

	*Total sample (N=10477) % (n)*	*CCurrent smokers (N=3327) % (n)*	*Firmer smokers (N=1124) % (n)*	*Never smokers (N=6026) % (n)*	*X^2^*	*p*
Bought e-cigarettes as gifts	23.73 (2486)	33.21 (1105)	32.03 (360)	16.94 (1021)	361.5	0.0001
Recommended e-cigarettes to others	33.26 (3485)	44.33 (1475)	41.19 (463)	25.67 (1547)	372.0	0.0001

### Multivariate analysis

Models 1–4 tested the association between smoking status and e-cigarette awareness and use. Table 4 presents the results.

**Table 4:** Logistic regression results of awareness and use of e-cigarettes

*Variables*	*Awareness of e-cigarettes*		*Use of e-cigarettes*	
	*Model 1 OR (95% CI)*	*Moilel 2 OR (95% CI)*	*Model 3 OR (95% CI)*	*Moilel 4 OR (95% CI)*
**Tobacco smoking status**					
Current smoker	3.26 (2.70-3.93)***		14.27 (12.24-16.64)***	
Tried to quit		4.78 (3.76-6.08)***		16.44 (14.02-19.26)***
Not tried to quit		1.57 (1.21-2.04)***		8.31 (6.72-10.27)***
Former smoker	1.94 (1.54-2.45)***	1.94 (1.54-2.45)***	13.34 (11.16-15.95)***	13.43 (11.23-16.06)***
Never smoker	1.00	1.00	1.00	1.00
**Perception of e-cigarettes**					
Not harmful to users			1.19 (1.04-1.36)*	1.21 (1.05-1.39)**
Harmful to users			1.00	1.00
No secondhand smoke harm			1.12 (0.97-1.28)	1.12 (0.98-1.29)
Secondhand smoke harm			1.00	1.00
Not addictive			1.82 (1.61-2.06)***	1.83 (1.62-2.06)***
Addictive			1.00	1.00
Safe replacement for cigarettes			0.997 (0.89-1.12)	1.01 (0.90-1.13)
Not a safe replacement for cigarettes			1.00	1.00
**Family member/friend use**					
Yes			3.44 (3.07-3.85)***	3.36 (2.99-3.77)***
No			1.00	1.00
Drinking alcohol					
Often			2.87 (2.29-3.60)***	2.86 (2.28-3.59)***
Occasionally			1.87 (1.56-2.24)***	1.86 (1.56-2.23)***
Seldom			1.32 (1.09-1.60)**	1.33 (1.09-1.61)**
Never			1.00	1.00
**Gender**					
Male	1.42 (1.24-1.62)“*	1.40 (1.22-1.60)***	1.70 (0.93-1.23)	1.05 (0.92-1.21)
Female	1.00	1.00	1.00	1.00
**Economic status**					
Very wealthy	5.55 (3.18-9.70)***	5.41 (3.09-9.46)***	3.44 (2.15-5.48)***	3.41 (2.13-5.45)***
Comfortable	4.50 (3.42-5.93)***	4.45 (3.37-5.87)***	1.51 (1.01-2.26)*	1.50 (0.998-2.25)*
Fair	3.66 (2.82-4.76)***	3.64 (2.80-4.74)***	1.02 (0.68-1.53)	1.02 (0.68-1.53)
Difficult	2.24 (1.64-3.07)***	2.23 (1.63-3.05)***	1.31 (0.84-2.06)	1.29 (0.82-2.03)
Very difficult	1.00	1.00	1.00	1.00
**Education level**					
Junior college	1.24 (1.03-1.49)***	1.25 (1.04-1.49)*	1.10 (0.94-1.29)	1.11 (0.95-1.29)
College	0.95 (0.79-1.13)	0.95 (0.80-1.14)	1.22 (1.04-1.43)*	1.22 (1.04-1.43)*
Postgraduate	0.82 (0.61-1.12)	0.84 (0.62-1.15)	1.53 (1.16-2.03)***	1.59 (1.20-2.11)***
High school or lower	1.00	1.00	1.00	1.00
**Marital status**					
Married	1.39 (1.10-1.76)**	1.34 (1.06-1.70)*	0.43 (0.35-0.52)***	0.41 (0.33-0.50)***
Never married	1.23 (1.00-1.52)*	1.19 (0.97-1.47)	0.38 (0.32-0.46)***	0.36 (0.30-0.43)***
Other	1.00	1.00	1.00	1.00
**Employment status**					
Full-time employment	1.12 (0.97-1.30)	1.11 (0.96-1.28)	1.11 (0.96-1.27)	1.08 (0.93-1.24)
Part-time employment	0.97 (0.82-1.14)	0.97 (0.83-1.14)	1.17 (0.998-1.37)	1.16 (0.99-1.37)
Unemployed	1.00	1.00	1.00	1.00
**Type and location of hukou (household registration)**					
Urban	0.98 (0.86-1.11)	0.98 (0.86-1.11)	0.73 (0.65-0.81)***	0.72 (0.65-0.81)***
Rural	1.00	1.00	1.00	1.00
Local	0.75 (0.66-0.86)***	0.77 (0.67-0.87)***	0.95 (0.84-1.07)	0.97 (0.86-1.09)
Migrant	1.00	1.00	1.00	1.00

* p<0.05

** p<0.01

*** p<0.001.

First, both current and former smokers had a higher odds ratio of knowing and using e-cigarettes than never smokers. By further breaking down the smoking status, we found that both the smokers who had tried to quit smoking and those who had not were more likely than never smokers to be aware of and to have used e-cigarettes at the time of the survey. More interestingly, current smokers who had tried to quit had a much higher odds ratio of knowing and using e-cigarettes than those who had not, suggesting a relationship between quitting smoking behavior and e-cigarette knowledge and use. Both Model 3 and Model 4 also showed that those who believed that e-cigarettes were not harmful for their health and not addictive were more likely to use e-cigarettes.

Second, as shown by other studies^29^, the family and peer influence on e-cigarette use was evident in our study as well. Seen for Model 4, the odds ratio for those with family members or friends using e-cigarettes was 3.36 times that of those who had no such family members or friends. Also, those with any degree of alcohol drinking were more likely to use e-cigarettes than those who never drank, confirming that concurrent use of other substances may be associated with e-cigarette use^30^ .

Third, men had a significantly higher probability of knowing about e-cigarettes than women, but their probability of using e-cigarettes was not necessarily higher. Less educated people were more likely to hear about e-cigarettes than better educated people but less likely to use them. Urban residents had a higher odds ratio of using e-cigarettes than rural residents. Married people with better economic status were more likely to learn about and use e-cigarettes.

Table 5 presents the results of the correlates of e-cigarette promotion. Both current and former smokers were more likely to buy e-cigarettes as gifts, and current smokers who had tried to quit smoking had a higher odds ratio of sending e-cigarettes as gifts, suggesting that e-cigarette gifting was due in part to the intention of sharing a perceived positive experience with friends. Current smokers also were more likely to recommend e-cigarettes to others. When separating current smokers by their quitting behavior, we found that current smokers, whether they had tried to quit or not, had a higher odds ratio of recommending e-cigarettes to others than never smokers, but those who had tried to quit were even more likely to recommend e-cigarettes to others. Those who believed that e-cigarettes were not harmful to users, caused no secondhand smoke harm, were not addictive, and were a safe replacement of cigarettes had higher odds ratios of e-cigarette gifting and recommendation.

**Table 5:** Logistic regression results of sending e-cigarettes as gifts and recommending e-cigarettes to others

*Variables*	*Sending e-cigarettes as gifts*		*Recommending e-cigarettes to others*	
	*Model 1 OR (95% CI)*	*Model 2 OR (95% CI)*	*Model 3 OR (95% CI)*	*Model 4 OR (95% CI)*
**Tobacco smoking status**					
Current smoker	1.34 (1.17-1.52)“*		1.26 (1.12-1.43)***	
Tried to quit		1.49 (1.30-1.71)***		1.45 (1.28-1.65)***
Not tried to quit		0.85 (0.68-1.07)		0.74 (0.60-0.91)***
Former smoker	1.32 (1.10-1.57)***	1.32 (1.11-1.57)***	1.16 (0.98-1.36)	1.16 (0.98-1.36)
Never smoker	1.00	1.00	1.00	1.00
**Perception of e-cigarettes**					
Not harmful to users	1.25 (1.09-1.43)***	1.26 (1.10-1.44)***	1.43 (1.26-1.62)***	1.45 (1.27-1.64)***
Harmful to users	1.00	1.00	1.00	1.00
No secondhand smoke harm	1.20 (1.05-1.38)**	1.20 (1.05-1.38)**	1.56 (1.38-1.77)***	1.57 (1.39-1.78)***
Secondhand smoke harm	1.00	1.00	1.00	1.00
Not addictive	1.67 (1.45-1.88)***	1.67 (1.48-1.88)***	2.01 (1.81-2.25)***	2.02 (1.81-2.26)***
Addictive	1.00	1.00	1.00	1.00
Safe replacement for cigarettes	0.79 (0.70-0.89)***	0.79 (0.70-0.89)***	0.66 (0.59-0.73)***	0.66 (0.59-0.74)***
Not a safe replacement for cigarettes	1.00	1.00	1.00	1.00
**Family member/friend use**					
Yes	5.65 (5.06-6.31)***	5.59 (5.00-6.25)***	4.24 (3.84-4.68)***	4.19 (3.79-4.62)***
No	1.00	1.00	1.00	1.00
**Drinking alcohol**					
Often	2.14 (1.70-2.69)***	2.13 (1.69-2.68)***	2.26 (1.82-2.81)***	2.25 (1.81-2.80)***
Occasionally	1.76 (1.50-2.06)***	1.76 (1.50-2.06)***	1.59(1.38-1.83)***	1.59 (1.38-1.83)***
Seldom	1.36 (1.15-1.60)***	1.36 (1.16-1.61)***	1.16 (1.01-1.34)*	1.17 (1.01-1.35)*
Never	1.00	1.00	1.00	1.00
**Gender**					
Male	0.88 (0.77-0.99)*	0.87 (0.76-0.98)*	0.92 (0.82-1.04)	0.91 (0.81-1.02)
Female	1.00	1.00	1.00	1.00
**Economic status**					
Very wealthy	2.80 (1.69-4.63)“*	2.79 (1.69-4.63)***	1.51 (0.95-2.39)	1.50 (0.94-2.37)
Comfortable	1.70 (1.11-2.60)*	1.70 (1.11-2.60)*	1.37 (0.95-1.97)	1.36 (0.94-1.97)
Fair	1.15 (0.75-1.75)	1.15 (0.75-1.75)	1.01 (0.70-1.45)	1.00 (0.69-1.44)
Difficult	1.11 (0.69-1.79)	1.10 (0.68-1.78)	1.13 (0.75-1.70)	1.11 (0.74-1.68)
Very difficult	1.00	1.00	1.00	1.00
**Education level**					
Junior college	1.18 (1.01-1.38)*	1.19 (1.01-1.39)*	1.21 (1.05-1.40)**	1.22 (1.05-1.40)**
College	1.36 (1.16-1.60)***	' 1.36 (1.16-1.60)***	1.30 (1.13-1.51)***	1.31 (1.13-1.52)***
Postgraduate	1.30 (0.97-1.73)	1.34 (1.00-1.78)*	1.13 (0.86-1.48)	1.16 (0.89-1.53)
High school or lower	1.00	1.00	1.00	1.00
**Marital status**					
Married	1.03 (0.83-1.26)	0.99 (0.81-1.22)	1.49 (1.22-1.82)***	1.44 (1.18-1.76)***
Never married	0.77 (0.63-0.93)**	0.75 (0.61-0.90)**	1.14 (0.95-1.37)	1.10 (0.92-1.32)
Other	1.00	1.00	1.00	1.00
**Employment status**					
Full-time employment	1.41 (1.23-1.62)***	1.39 (1.22-1.60)***	1.35 (1.19-1.52)***	1.33 (1.17-1.50)***
Part-time employment	1.41 (1.21-1.64)***	1.41 (1.20-1.64)***	1.26 (1.10-1.45)***	1.26 (1.10-1.45)***
Unemployed	1.00	1.00	1.00	1.00
**Type and location of hukou (household registration)**					
Urban	0.84 (0.75-0.93)***	0.84 (0.75-0.93)***	0.89 (0.80-0.98)*	0.89 (0.80-0.98)*
Rural	1.00	1.00	1.00	1.00
Local	0.90 (0.80-1.02)	0.91 (0.81-1.03)	0.92 (0.82-1.03)	0.93 (0.84-1.04)
Migrant	1.00	1.00	1.00	1.00

* p<0.05

** p<0.01

*** p<0.001.

## DISCUSSION

This study reveals some important patterns of e-cigarette use among young adults in China. Specifically, we examined the awareness and perception of e-cigarettes and the determinants of e-cigarette use by current, former, and never smokers. Moreover, we explored the e-cigarette gifting behavior, which has long been a tradition among Chinese conventional smokers.

By the time of our survey, e-cigarettes were extensively known by young adults in China, and nearly a quarter of all respondents had used them. However, the perceptions of the health impacts of e-cigarettes were rather divided, and a fairly large number of respondents had no idea about e-cigarette health impacts. As e-cigarettes become increasingly accessible and prevalent among young adults in China, the health impacts of e-cigarette use need to be further examined by researchers so that more scientific information on e-cigarettes can be made available to the public.

Our study also found that there is an association between smoking behavior and e-cigarette perception and use. First, both current and former smokers in our survey tended to hold a more positive attitude toward e-cigarettes as they were more inclined to understate the harm of e-cigarettes than never smokers.

Second, while about 50% of smokers had used e-cigarettes, much higher than never smokers, they also were more likely to believe that e-cigarettes were a safe replacement for conventional cigarettes than were never smokers, and almost half of current smokers used e-cigarettes to help them quit smoking. As our multivariate tests showed, current smokers who had tried to quit were much more likely to use e-cigarettes than never smokers. Our finding differs from that of the little existing research on e-cigarettes in China. While Xiao et al.^5^ found that Chinese youth used e-cigarettes as a tobacco product rather than an aid to smoking cessation, our findings suggest that e-cigarettes might be used as a tool by young adult smokers to quit smoking, although caution is needed as it is unknown whether smokers first tried to quit or used an e-cigarette. Whether this outcome materializes depends on more research on whether e-cigarette use may help with smoking reduction or abstinence. Several important studies did demonstrate the effect^31-33^ although others raised doubt^34^. Since the Xiao et al. study focused on middle school students in China while ours on young adults, the difference in findings with regard to e-cigarette use and smoking behavior might be due to the fact that young adults have longer smoking history and are more concerned about smoking cessation.

Third, the gifting culture was evident among e-cigarette users as it is among conventional smokers, and smokers, especially current smokers who had tried to quit, than never smokers, were more willing to share e-cigarettes with others. As a social norm of smoking, cigarette gifting is a strong barrier for China to have effective tobacco control^27^. While the evidence of e-cigarette impact on smoking cessation is inadequate, if e-cigarette is proved to be associated with quitting smoking attempts, e-cigarette gifting among smokers at least helps spread the norm that cigarette smoking is harmful.

### Limitations

A major limitation of this study is that the findings are based on a cross-sectional and mobile app survey, and thus we are not able to test causality and the sample does not allow us to make generalizations. Nonetheless, the present study paves the way for future research that will aim at a more representative sample and adopt a longitudinal design to track e-cigarette use and perceptions, and smoking behavior over time.

## CONCLUSIONS

Our study shows that e-cigarettes have gained popularity among young adults in China, with 88.40% of the respondents being aware of e-cigarettes and 24.45% having used them. Also, smokers were more likely to perceive e-cigarettes as less harmful and to have used e-cigarettes. Smokers who had tried to quit had a higher tendency to use e-cigarettes. Finally, we have identified an e-cigarette gifting culture among smokers and especially those who had tried to quit, as they were more likely than never smokers to have shared e-cigarettes with others, by either giving them as gifts or recommending them to others.

Despite the increasing popularity of e-cigarettes in China, without hard evidence on e-cigarette harm to health and its effect on smoking cessation, policymakers in China face the dilemma of either promoting e-cigarettes as an alternative to cigarettes or banning both completely. Recently, China Tobacco and China E-cigarette Chamber of Commerce both issued policies to ban e-cigarette sales to youths under 18 years old. Also, e-cigarette smoking in public places has been banned in cities, including Beijing and Hong Kong, and in high-speed trains. While we agree that necessary regulatory steps need to be taken to avoid unnecessary harm from e-cigarettes, our research suggests that young smokers at least considered e-cigarettes as aiding smoking cessation and, therefore, before more concrete evidence on the relationship between e-cigarette use and smoking cessation becomes available, a more modest vision toward a national regulatory framework needs to be taken.

## CONFLICTS OF INTEREST

The authors have completed and submitted an ICMJE form for disclosure of potential conflicts of interest and they declare that they have no competing interests, financial or otherwise, related to the current work. X Wang, X Zhang, X Xu and Y Gao report grants from the Bill and Melinda Gates Foundation, during the conduct of the study.
